# Supplementary material for: DNA Damage in Oocytes Induces a Switch of the Quality Control Factor TAp63α from Dimer to Tetramer
Source: Cell. 2011 Feb 18;144(4):566–76. doi: 10.1016/j.cell.2011.01.013 (PMC3087504; doi:10.1016/j.cell.2011.01.013)
Supplement: Table S1. The Identification of TAp63α in a Dimeric State by Analytical Ultracentrifugation, Related to Figure 1 [file mmc1.pdf]

Protein	M_W (kDa)	$M_{W, fit}$ (kDa), [n*]
MBP-TAp63α	116.0	230.0#, [2.0]<br>249.5$, [2.2]
MBP-TAp63αFTL	115.9	336.6#, [2.9]<br>389.3$, [3.4]
MBP-TAp63γ	94.2	308.0#, [3.3]<br>327.2$, [3.5]

* theoretical number of monomers

#calculated using ULTRASPIN

$calculated using HETEROANALYSIS
