# Supplementary material for: DNA Damage in Oocytes Induces a Switch of the Quality Control Factor TAp63α from Dimer to Tetramer
Source: Cell. 2011 Feb 18;144(4):566–76. doi: 10.1016/j.cell.2011.01.013 (PMC3087504; doi:10.1016/j.cell.2011.01.013)
Supplement: Document S1. Article Plus Supplemental Information [file mmc2.pdf]

Cell

# DNA Damage in Oocytes Induces a Switch of the Quality Control Factor TAp63α from Dimer to Tetramer

Gregor B. Deutsch,[1,12] Elisabeth M. Zielonka,[2,3,12] Daniel Coutandin,[1,12] Tobias A. Weber,[1] Birgit Schäfer,[1] Jens Hannewald,[4] Laura M. Luh,[1] Florian G. Durst,[1] Mohamed Ibrahim,[5] Jan Hoffmann,[6] Frank H. Niesen,[8] Aycan Sentürk,[7] Hana Kunkel,[10] Bernd Brutschy,[6] Enrico Schleiff,[5] Stefan Knapp,[8,9] Amparo Acker-Palmer,[7] Manuel Grez,[10] Frank McKeon,[2,3,11] and Volker Dötsch[1,*]
[1]Institute of Biophysical Chemistry and Center for Biomolecular Magnetic Resonance, Goethe University, Frankfurt 60438, Germany
[2]ISIS, University of Strasbourg, Strasbourg 67000, France
[3]The Genome Institute of Singapore, A-STAR, Singapore 138672, Singapore
[4]MS-DTB-C Protein Purification, Merck KGaA, Darmstadt 64293, Germany
[5]Institute of Molecular and Cell Biology of Plants
[6]Institute of Physical and Theoretical Chemistry
[7]Frankfurt Institute for Molecular Life Sciences (FMLS) and Institute of Cell Biology and Neuroscience
Goethe University, Frankfurt 60438, Germany
[8]Nuffield Department of Medicine
[9]Department of Clinical Pharmacology
Structural Genomics Consortium, Old Road Campus Research Building, Oxford University, Oxford OX3 7DQ, UK
[10]Georg-Speyer Haus, Frankfurt 60596, Germany
[11]Department of Cell Biology, Harvard Medical School, Boston, MA 02115, USA
[12]These authors contributed equally to this work
*Correspondence: vdoetsch@em.uni-frankfurt.de
DOI 10.1016/j.cell.2011.01.013

## SUMMARY

TAp63α, a homolog of the p53 tumor suppressor, is a quality control factor in the female germline. Remarkably, already undamaged oocytes express high levels of the protein, suggesting that TAp63α's activity is under tight control of an inhibitory mechanism. Biochemical studies have proposed that inhibition requires the C-terminal transactivation inhibitory domain. However, the structural mechanism of TAp63α inhibition remains unknown. Here, we show that TAp63α is kept in an inactive dimeric state. We reveal that relief of inhibition leads to tetramer formation with ~20-fold higher DNA affinity. In vivo, phosphorylation-triggered tetramerization of TAp63α is not reversible by dephosphorylation. Furthermore, we show that a helix in the oligomerization domain of p63 is crucial for tetramer stabilization and competes with the transactivation domain for the same binding site. Our results demonstrate how TAp63α is inhibited by complex domain-domain interactions that provide the basis for regulating quality control in oocytes.

## INTRODUCTION

In mammals, the family of the p53 tumor suppressor contains two additional members, p73 and p63 (Kaghad et al., 1997; Schmale and Bamberger, 1997; Senoo et al., 1998; Trink et al., 1998; Yang et al., 1998). Originally, their discovery had sparked the speculation that tumor suppression might be achieved by an entire network of p53-like factors. However, detailed studies have revealed that both p53 homologs serve important developmental functions (Mills et al., 1999; Yang et al., 1999, 2000). p73 knockout mice suffer from hippocampal dysgenesis, chronic infections and inflammation, as well as abnormalities in pheromone sensory pathways. Loss of p63 results in even more severe defects, including limb truncations, lack of a multilayered skin, and other epithelial structures. These phenotypes led to the identification of six p63-related syndromes in humans that are characterized by deformation of the limbs and/or skin abnormalities (Celli et al., 1999; McGrath et al., 2001). In contrast to these clear developmental functions the question if both proteins also act as tumor suppressors is still debated.

The investigation of the physiological role of both proteins is further complicated by the existence of many different isoforms of p63 and p73 and potentially by the formation of mixed oligomers between both proteins (Coutandin et al., 2009; Joerger et al., 2009) (Figure 1A). Isoforms are created by combining several different C-terminal splice variants (De Laurenzi et al., 1999; Kaghad et al., 1997; Yang et al., 1998) with two different promoters that produce isoforms either with (TA isoforms) or without (ΔN isoforms) the N-terminal transactivation domain (Yang et al., 1998).

In the case of p63, detailed analysis has started to reveal the physiological functions of some isoforms. ΔNp63α, the isoform produced by combination of the second promoter with the longest C-terminal splice variant, plays an important role in the

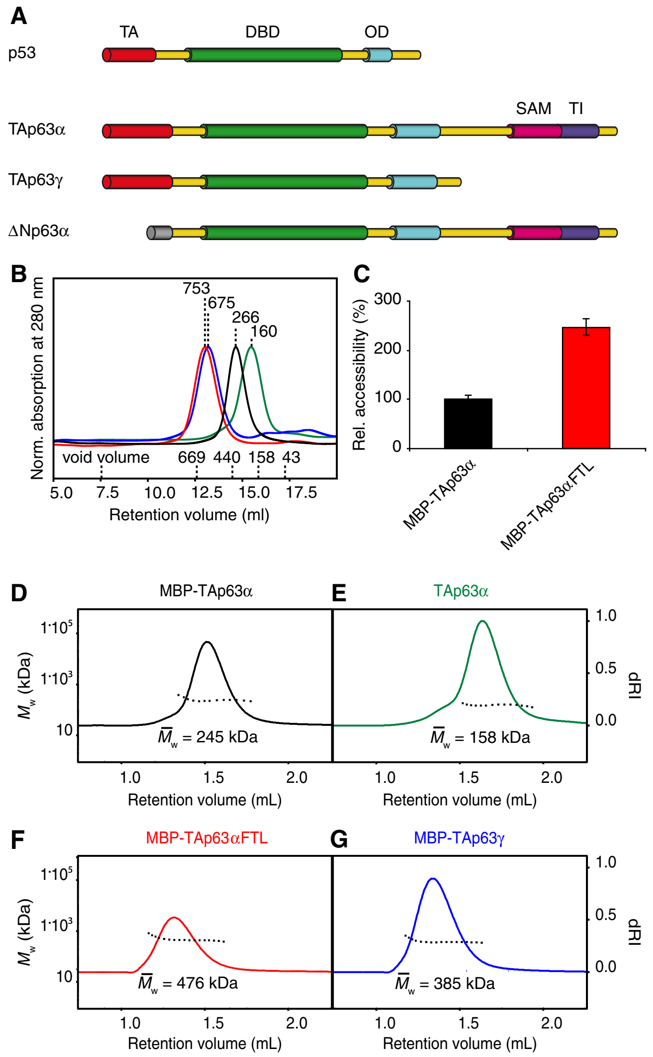


**Figure 1. TAp63α Is Kept in an Inactive Dimeric State**

(A) Comparison of the domain structure of p53, TAp63α, TAp63γ, and ΔNp63α showing the transactivation (TA) domain, DNA binding domain (DBD), oligomerization domain (OD), sterile α-motive (SAM) domain, and transactivation inhibitory (TI) domain.

(B) SEC chromatogram of TAp63α (green), MBP-TAp63α (black), MBP-TAp63αFTL (red), and MBP-TAp63γ (blue) purified from *E. coli*. Apparent molecular weights are indicated. Void volume and elution volumes of globular standard proteins with corresponding molecular weights are shown on the x axis.

(C) Bar diagram showing relative accessibilities of cysteines in MBP-TAp63α (black) and MBP-TAp63αFTL (red) as monitored by binding of a maleimide-biotin conjugate. The value of MBP-TAp63α is set to 100%. Error bars show standard deviation.

(D–G) Change of differential refractive index (dRI, solid line) and of the molecular weight of the protein peak ($M_W$, dotted line) in SEC-MALS analysis of MBP-TAp63α (D), TAp63α (E), MBP-TAp63αFTL, (F) and MBP-TAp63γ (G). Calculated molecular weights are displayed.

See also Figure S1 and Table S1.

development of stratified epithelial tissues including skin by maintaining a stem cell population in the basal layer (Senoo et al., 2007). Inactivation of this isoform seems to be responsible for the severe developmental defects seen in the knockout mice as well as in human patients with p63 mutations. In contrast, TAp63α, the isoform containing the long α C terminus and the full N-terminal transactivation domain, serves a quite different function. It is found in oocytes where it acts as a quality control factor that monitors the genetic stability of these cells (Suh et al., 2006). TAp63α expression in murine oocytes can be detected from day E18.5 on and at day P5, when murine oocytes are arrested in prophase of meiosis I, all oocytes show strong nuclear expression. After oocytes are recruited for ovulation, TAp63α expression is lost. Previous experiments have shown that γ-irradiation results in phosphorylation of TAp63α followed by the elimination of all premature oocytes while mature ones that do not express p63 are not affected (Suh et al., 2006).

Due to its ability to induce cell death the activity of TAp63α must be regulated very tightly. In contrast to p53 that is kept at very low concentrations in nonstressed cells and only increases when triggered by oncogenic signals (Brooks and Gu, 2006; Wade et al., 2010), TAp63α reaches high expression levels already in nonstressed oocytes (Suh et al., 2006). This observation has suggested that the activity of TAp63α must be regulated by keeping it in an inactive state. In cell culture-based transactivation experiments TAp63α indeed showed only a very low transcriptional activity. By deletion mutagenesis we have revealed in previous experiments that the last 70 amino acids of TAp63α act as a transactivation inhibitory domain (TID). Deletion or mutation of this TI domain increases TAp63α's low transcriptional activity severalfold (Serber et al., 2002; Straub et al., 2010; Yang et al., 1998). The exact mechanism of how the TID inhibits the activity of TAp63α, however, remained unknown.

Here, we show both in vitro and in vivo that TAp63α is kept in a closed dimeric conformation in nonstressed oocytes while γ-irradiation-triggered phosphorylation promotes the formation of active tetramers. The active state of TAp63α is stabilized by a special structure of its tetramerization domain, making the activation process essentially irreversible.

## RESULTS

### TAp63α Forms a Closed Dimeric Conformation

To characterize the inhibitory mechanism of the TID we expressed murine TAp63α in *Escherichia coli*. Surprisingly, size exclusion chromatography (SEC) analysis of purified TAp63α suggested that it forms dimers instead of the expected tetramers (Figure 1B). All mammalian members of the p53 protein family use a highly conserved oligomerization domain (OD) to form tetramers that were shown to be the active state (Chan et al., 2004; Chene, 2001; Jeffrey et al., 1995; Lee et al., 1994). The concentration of TAp63α used in the SEC experiment was orders of magnitude higher (3–15 μM) than the $K_D$ for tetramerization of p63 (12 nM) (Brandt et al., 2009), excluding the possibility that simple dilution can explain the absence of tetramers. This result implies that TAp63α adopts a dimeric, inactive conformation and further suggests that activation of p63 may be linked to the formation of tetramers. To address this question, we performed SEC analysis with the transcriptionally active TAp63γ isoform that lacks the TID. Obtaining soluble TAp63γ required its expression as an N-terminal maltose-binding-protein (MBP) fusion. For

comparability we also expressed TAp63α fused to MBP. SEC analysis of MBP-TAp63γ showed that it behaves as a significantly larger protein than MBP-TAp63α despite the fact that it contains 193 amino acids less per monomer (Figure 1B).

In a previous alanine scanning experiment of the TID, we have found that the triple mutant F605A T606A L607A (TAp63αFTL) shows high transcriptional activity suggesting that this mutation destroys the inhibitory function of the TID (Straub et al., 2010). SEC analysis showed that MBP-TAp63αFTL also behaves as a much larger protein than MBP-TAp63α with a retention volume similar to MBP-TAp63γ (Figure 1B).

The classification of TAp63α as a dimer was based on a calibration of the SEC column with compact globular proteins. The predicted molecular weights of 160 kDa for TAp63α and 266 kDa for MBP-TAp63α closely resemble the theoretical values for the dimers of 150 and 232 kDa, respectively. Estimation of the molecular weights of MBP-TAp63αFTL and MBP-TAp63γ, however, resulted in values exceeding the theoretical ones for the assumed tetramers by far. These findings could be explained either if the active forms adopt an oligomeric state higher than tetrameric, if the protein aggregates or if the conformation of the tetramers deviates from a globular fold. To investigate the oligomeric state of all proteins by determining their mass independently of their shape, we used multiangle light scattering (MALS) analysis. The results shown in Figures 1D–1G confirm that TAp63α and MBP-TAp63α form dimers, while MBP-TAp63αFTL and MBP-TAp63γ exist as tetramers. The homogeneity of the SEC peaks demonstrated by MALS analysis further excludes the possibility that the proteins aggregate. The absence of aggregation and the formation of specific oligomeric states are further supported by crosslinking experiments (see Figures S1A–S1D available online), by mass spectrometry (LILBID) analysis (Figure S1E) and sedimentation equilibrium analytical ultracentrifugation (Figure S1F and Table S1). The combination of all these data suggests that the deviation of the apparent molecular weight from the calculated one originates from the formation of an open, nonglobular conformation. To further investigate this hypothesis we performed a cysteine accessibility assay that demonstrated that cysteines can be more easily chemically modified in the MBP-TAp63αFTL mutant than in the nonmutated form (Figure 1C; Figure S1I). In addition, the formation of an open conformation was supported by the analysis of sedimentation velocity ultracentrifugation experiments (Figures S1G and S1H).

## Phosphorylation Triggers the Formation of Active TAp63α Tetramers In Vivo

In mice, expression of TAp63α can be detected from embryonic day 18.5 on with all oocytes showing strong expression at day P5 when murine oocytes are arrested in prophase of meiosis I (dictyate arrest) (Suh et al., 2006). p63 expression is maintained at a high level until oocytes are recruited for ovulation. DNA damage during this time triggers activation of p63 and destruction of the oocytes. To investigate if our results obtained in vitro can explain the behavior of TAp63α in oocytes, we analyzed the oligomeric state of TAp63α in 5-day-old mice by SEC. Figure 2A shows that γ-irradiation of mice with 0.52 Gy triggers phosphorylation of TAp63α and leads to a reduction of its concentration relative to nonirradiated oocytes. Analysis of the SEC fractions of samples obtained from nonirradiated mice revealed a strong signal in the dimer fraction (1.55 ml) and no detectable signal in the tetramer fraction (1.3 ml, calibrated with bacterially expressed p63 isoforms; Figures S2A–S2C and Figures 2A–2C). In contrast, samples obtained from irradiated mice showed a significant signal in the tetramer fraction (Figures 2A, 2D, and 2E). The in vivo concentration of tetrameric TAp63α in irradiated oocytes is expected to be significantly higher than seen in the SEC experiments since oocyte lysis and SEC analysis lead to a minimum 10-fold dilution of the sample. We confirmed that dilution shifts the equilibrium toward dimers by reducing the concentration of bacterially expressed MBP-TAp63γ in SEC experiments (Figures S2C–S2G). While virtually exclusively tetrameric at a concentration of 3–15 μM, MBP-TAp63γ displays an almost equal distribution between dimers and tetramers at 30 nM (Figure S2). These results indicate that TAp63α in nonstressed oocytes is kept in a dimeric and closed conformation and that DNA damage triggers the formation of tetramers in vivo. The significantly higher concentration of TAp63α in nonirradiated versus irradiated cells further suggests that the

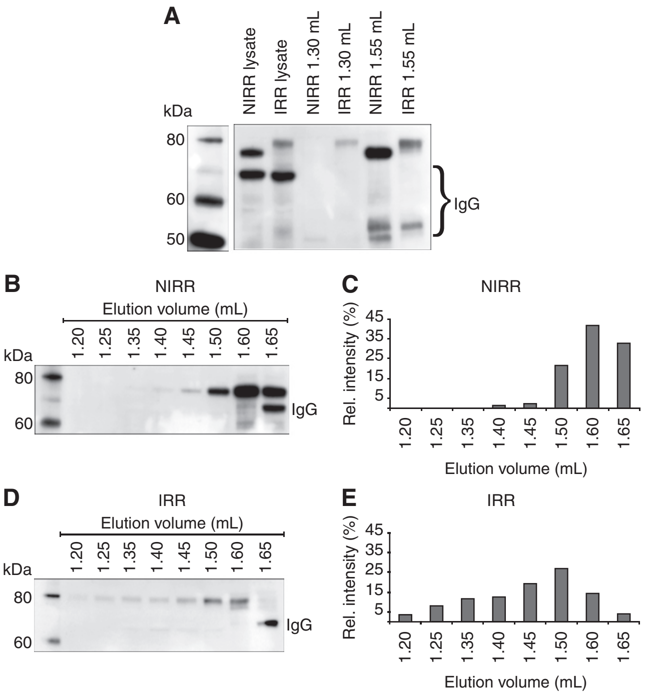


**Figure 2. Activation of TAp63α by Phosphorylation in Oocytes Leads to Tetramerization**

(A) Western blot of murine oocyte samples. p63 signals in the lysate of murine oocytes, SEC elution fractions at 1.3 ml (tetrameric protein) and 1.55 ml (dimeric protein) for both nonirradiated (NIRR) and γ-irradiated (IRR) oocytes are displayed.

(B) Western blot of SEC elution fractions from 1.2 to 1.65 ml of NIRR ovary lysate.

(C) Bar diagram showing relative p63 signal intensities of the western blot shown in (B). The sum of the intensities of all fractions was set to 100%.

(D and E) Corresponding data and analysis for IRR ovary lysate.

See also Figure S2.

formation of tetramers is not suppressed by keeping the intracellular concentration low (as it is discussed for p53) but actively by domain-domain interactions involving the TID.

## Tetramerization Increases the DNA Binding Affinity

We next investigated the functional consequences of tetramer formation. Previous experiments in oocytes have demonstrated that phosphorylation increases TAp63α's DNA binding affinity ~20-fold (Suh et al., 2006). To investigate if this increase in DNA affinity can be explained by the formation of tetramers, we measured $K_D$ values for the binding of MBP-TAp63α, MBP-TAp63αFTL, and MBP-TAp63γ to the p21 promoter response element using fluorescence anisotropy. Figures 3A–3C (and Figures S3A and S3B) reveal that dimeric MBP-TAp63α binds the p21 promoter response element with a $K_D$ of 7.51 ± 1.32 μM. For tetrameric MBP-TAp63αFTL and MBP-TAp63γ, $K_D$ values of 0.38 ± 0.04 μM and 0.34 ± 0.08 μM were measured, respectively. These measurements demonstrate that the change from a closed dimeric to an open tetrameric state increases the DNA binding affinity by ~20-fold, similar to the studies carried out in oocytes.

## Phosphorylation Is Not Required for Maintaining the Tetrameric State

Previous investigations have revealed that treatment of phosphorylated TAp63α with λ-phosphatase does not decrease the DNA binding affinity to its original value (Suh et al., 2006). This result suggests that phosphorylation serves as a trigger for the activation process but is not essential to maintain the active state. Since the active state is the tetramer, dephosphorylation should not affect the oligomerization equilibrium. To investigate the influence of the phosphorylation status on the oligomeric state, we performed SEC analysis of λ-phosphatase-treated TAp63α obtained from irradiated oocytes. The dephosphorylated sample indeed showed a high percentage of tetramers and only a relatively small increase in the dimer concentration compared with phosphorylated TAp63α (Figures 3D–3H). In vitro control experiments showed that λ-phosphatase treatment leads to complete dephosphorylation (Figures S3C and S3D). This result suggests that tetramer formation constitutes an almost irreversible activation switch triggered by phosphorylation. Recently, we and others have discovered that p63 contains an additional helix within its oligomerization domain (OD) that is not present in the p53 OD (Coutandin et al., 2009; Joerger et al., 2009). This second helix stabilizes the tetramer by reaching across the tetrameric interface. Since this helix must adopt a different conformation or orientation within the closed dimeric state, it might be the element that locks TAp63α in its tetrameric form after phosphorylation induced activation. This model is supported by the observation that deletion of this helix renders the active-state mimetic mutant TAp63αFTL dimeric while deletion had no effect on TAp63α (Figure 4). Furthermore, transactivation assays revealed that deletion of the second helix significantly reduced the transcriptional activity (Figure S4).

## The Tetramerization Domain Is Essential for Forming the Closed Conformation

This model of regulating the activity of p63 by controlling the oligomerization state assigns a pivotal role to the tetramerization domain (as TD we define the OD with the second helix). The structure of the TD is a dimer of dimers (Jeffrey et al., 1995; Lee et al., 1994). The most obvious model would explain the

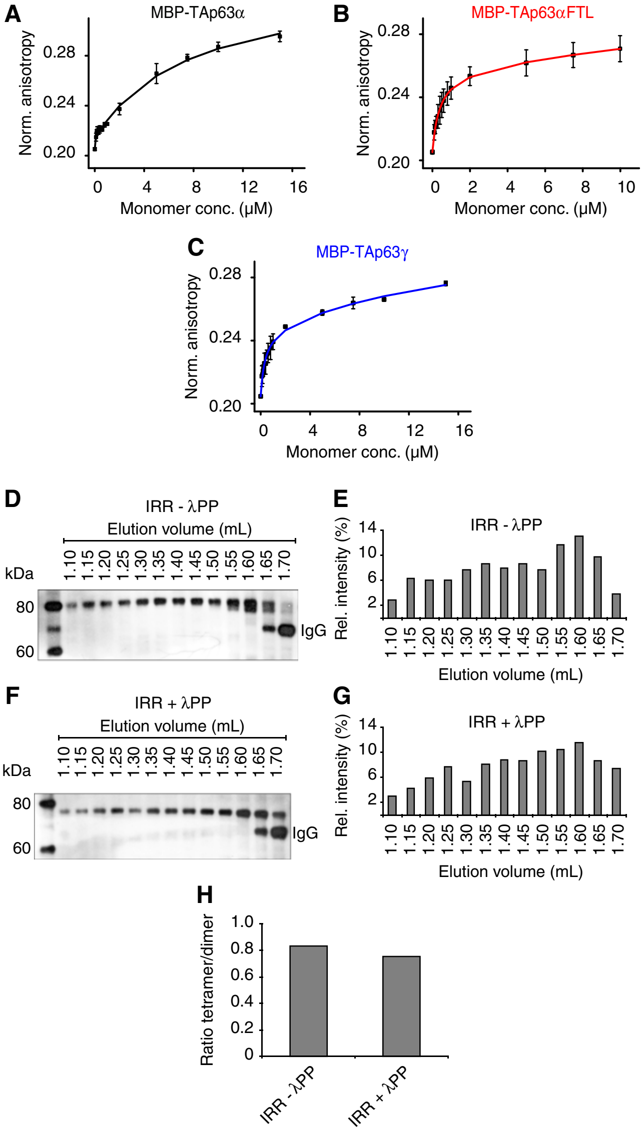


**Figure 3. Functional Consequences of Tetramer Formation**

(A–C) DNA-binding of MBP-TAp63α (A), MBP-TAp63αFTL (B), and MBP-TAp63γ (C) from *E. coli* to the p21 response element measured by fluorescence anisotropy. Solid lines show the fit to a two-binding-site model (see Supplemental Information). Error bars show standard deviation.

(D) Western blot of SEC elution fractions between 1.1 and 1.7 ml of IRR ovary lysate without λ-phosphatase treatment.

(E) Bar diagram representing the relative p63 intensities of the western blot shown in (D).

(F and G) Corresponding western blot and bar diagram for IRR lysate with λ-phosphatase treatment are shown in (F) and (G), respectively.

(H) Bar diagram showing ratios between the sum of the signal intensities of tetramer (1.10–1.40 ml) and dimer (1.45–1.70 ml) fractions of SEC elution fractions of ovary lysate without (IRR – λPP) and with (IRR + λPP) λ-phosphatase treatment.

See also Figure S3.

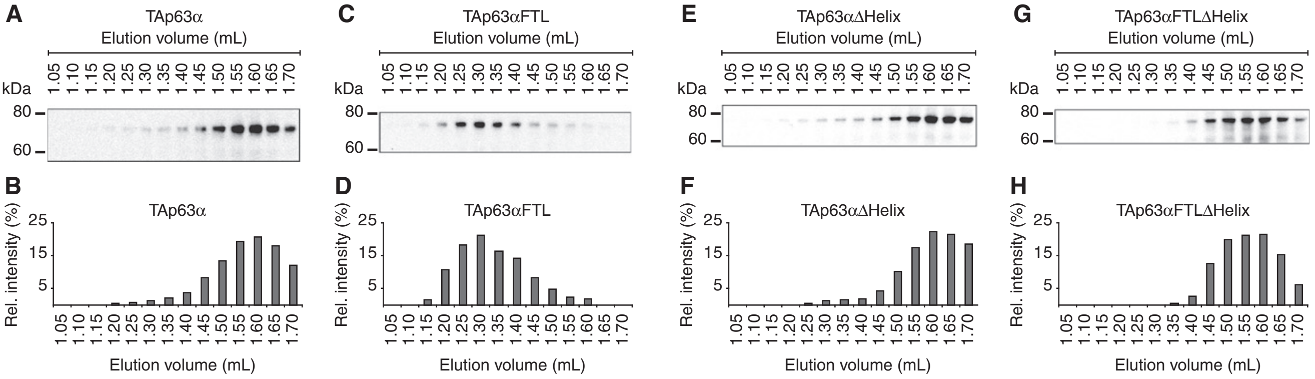


**Figure 4. The Second Helix of the TD Is Essential for Tetramer Formation**

(A) Western blot of SEC elution fractions between 1.05 and 1.70 ml of TAp63α expressed in rabbit reticulocyte lysate (RRL) using an anti-myc antibody.

(B) Bar diagram showing the relative p63 intensities of the western blot shown in (A). Corresponding data and analysis of TAp63αFTL, TAp63αΔHelix, and TAp63αFTLΔHelix are shown in (C and D), (E and F), and (G and H), respectively.

See also Figure S4.

inhibition of TAp63α by selective blocking of the tetramerization interface without affecting the dimerization interface. To probe the importance of the tetramerization interface we mutated Met374 to Gln and Ile378 to Arg (TAp63αMI). Mutations of analogs' position in p53 have been reported to trigger dimerization (Davison et al., 2001) (Figures 5A and 5B). These mutations increased the transcriptional activity significantly, reaching 55% activity of wild-type TAp63γ (Figure 5E), similar to the activity of dimeric p53 and TAp63γ mutants (Davison et al., 2001; Straub et al., 2010). Previous GST pull-down assays had shown that the TID and the TA domain interact with each other (Serber et al., 2002; Straub et al., 2010). In pull-down assays with an external TI domain the TAp63αMI mutant was effectively pulled down, in contrast to wild-type TAp63α (Figures 5F and 5G), suggesting that TAp63αMI forms an open conformation in which the TA is accessible for interaction. Consequently, this result predicts that the TID in TAp63αMI should also be accessible for interaction with an external TA domain. Indeed, this assumption was confirmed in pull-down experiments (Figures 5H and 5I). Taken together, these results demonstrate that this double mutation induces an open conformation by disrupting the inhibitory mechanism mediated by the TID.

Met374 and Ile378 are located in the center of the tetramerization interface. We also mutated Leu384 and Met385, located at its edge, to Ala (Figures 5A, 5C, and 5D). The L384A and L384A/M385A mutants (TAp63αL and TAp63αLM) showed very low activity in transactivation assays and no interaction in pull-down experiments with an external TID or TA domain (Figures 5E–5I), demonstrating that only mutations in the central region of the tetramerization domain disrupt the inhibitory mechanism by creating an open dimeric form (Figure S5).

An attractive model of the inhibitory mechanism would explain the formation of dimeric TAp63α by direct interaction of the TID with the tetramerization interface. In principle, NMR titration experiments would allow a direct mapping of the binding site. However, the p63 OD is tetrameric at concentrations typically used for NMR (even without the second helix) rendering the tetramerization interface inaccessible for a potential interaction with the TID. Consequently, NMR titrations of the p63 OD with a peptide derived from the TID (601–616) containing the FTL motif did not show any interaction. Interestingly, titration experiments of the p73 OD known to exist as a mixture of dimers and tetramers (Coutandin et al., 2009) with the p63 TID peptide resulted in the disappearance of all peaks by the formation of soluble aggregates. Repeating this titration with the p73 TD that forms stable tetramers did not show any interaction (data not shown). Although these experiments are quite indirect and involve mixing of p63 and p73 domains, they suggest that the p63 TID can interact with the p73 OD, but not with the p73 TD, the difference being that the p73 OD exists in an equilibrium with dimeric forms with an accessible tetramerization interface.

## The N-Terminal Transactivation Domain Binds to the OD of p63

Based on previous pull-down experiments, we had suggested that formation of the closed state of TAp63α involves both the N-terminal transactivation domain (TA) and the C-terminal TID (Serber et al., 2002). To further investigate the importance of the TA domain for the formation of the closed conformation, we performed SEC analysis of ΔNp63α, a natural occurring isoform lacking the first 45 amino acids (Yang et al., 1998). As shown in Figure 6A, ΔNp63α is tetrameric demonstrating that the simultaneous presence of both the TA and the TI domains is necessary for the formation of a closed, dimeric conformation.

To test whether the OD can interact with the TA domain we titrated the p63 OD with peptides derived from the TA1 (9–32) and TA2 (49–78) regions of the transactivation domain (Burge et al., 2009). While the TA2 peptide did not interact, titrations with the TA1 peptide showed strong chemical shift perturbations (CSP) in the fast exchange regime (Figures 6B, 6C, and 6F). Mapping these CSPs onto the OD structure revealed that the binding site for this peptide overlaps with the location of the second helix within the TD (Figure 6E). This result predicts that the TA1 peptide should not interact with the TD of p63 which

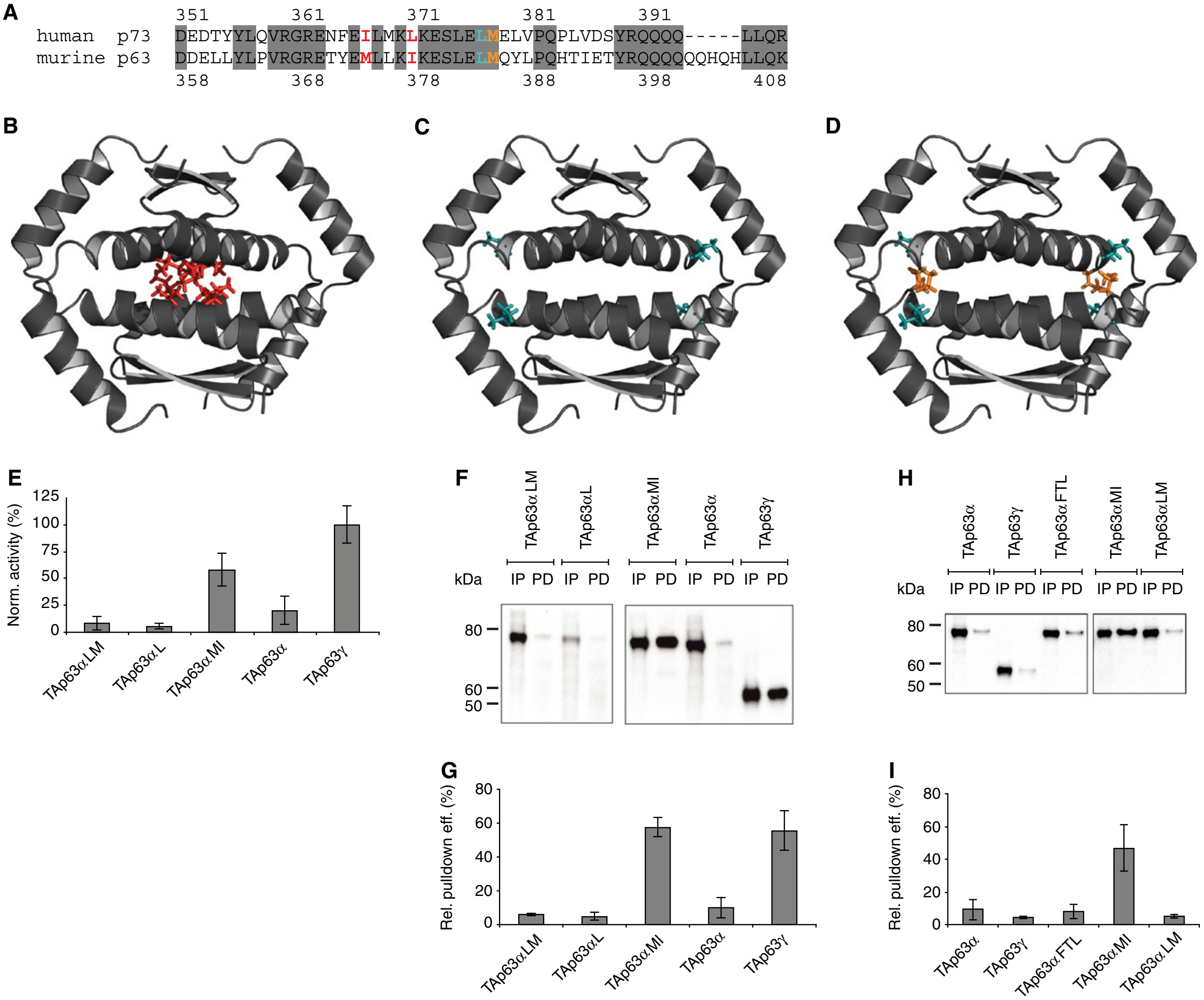


**Figure 5. The TD of TAp63α Plays an Essential Role in Maintaining the Inhibited Dimeric State**

(A) Sequence alignment of the TDs of human p73 and murine p63. Based on the structure of human p73 the structure of murine p63 was modeled. Conserved regions are indicated by gray boxes. Amino acids that were mutated in p63 are shown in red, cyan, and orange.

(B–D) Structure of the human p73 TD (PDB accession code: 2KBY). Side chains of residues that are homologous to the amino acids mutated in p63 are shown in the same colors as in (A). (B) shows mutations in TAp63αMI, (C) shows mutations in TAp63αL, and (D) shows mutations in TAp63αLM.

(E) Transcriptional activities on the p21 promoter in SAOS2 cells normalized to the protein concentration of TAp63αLM, TAp63αL, TAp63αMI, TAp63α, and TAp63γ. Error bars show standard deviation.

(F) Western blot of pull-down experiments with TAp63αLM, TAp63αL, TAp63αMI, TAp63α, and TAp63γ from RRL using immobilized TID. Input (IP) and pull-down (PD) are shown for each protein.

(G) Bar diagram showing the quantitative analysis of the pull-down experiments in (F). Error bars show standard deviation.

(H and I) Western blot (H) and corresponding bar diagram (I) showing the quantitative analysis of pull-down experiments with TAp63α, TAp63γ, TAp63αFTL, TAp63αMI, and TAp63αLM from RRL using immobilized TA domain. Error bars show standard deviation.

See also Figure S5.

contains the second helix. Repeating the titration experiments with the p63 TD indeed showed only very small chemical shift changes (Figure 6B), suggesting that both the second helix of the TD and the TA1 peptide compete for the same binding site.

The N-terminal transactivation domain of p53 contains three important residues, Phe19, Trp23, and Leu26, that are known to be involved in binding transcriptional coactivators and Mdm2 (Horikoshi et al., 1995; Kussie et al., 1996; Lu and Levine, 1995; Thut et al., 1995). The crystal structure of a peptide derived from the p53 transactivation domain in complex with Mdm2 showed that these three amino acids form one face of a helix that is deeply buried in a hydrophobic pocket of Mdm2. All three amino acids are conserved in p63. We hypothesized that if binding of the N-terminal transactivation domain to the OD

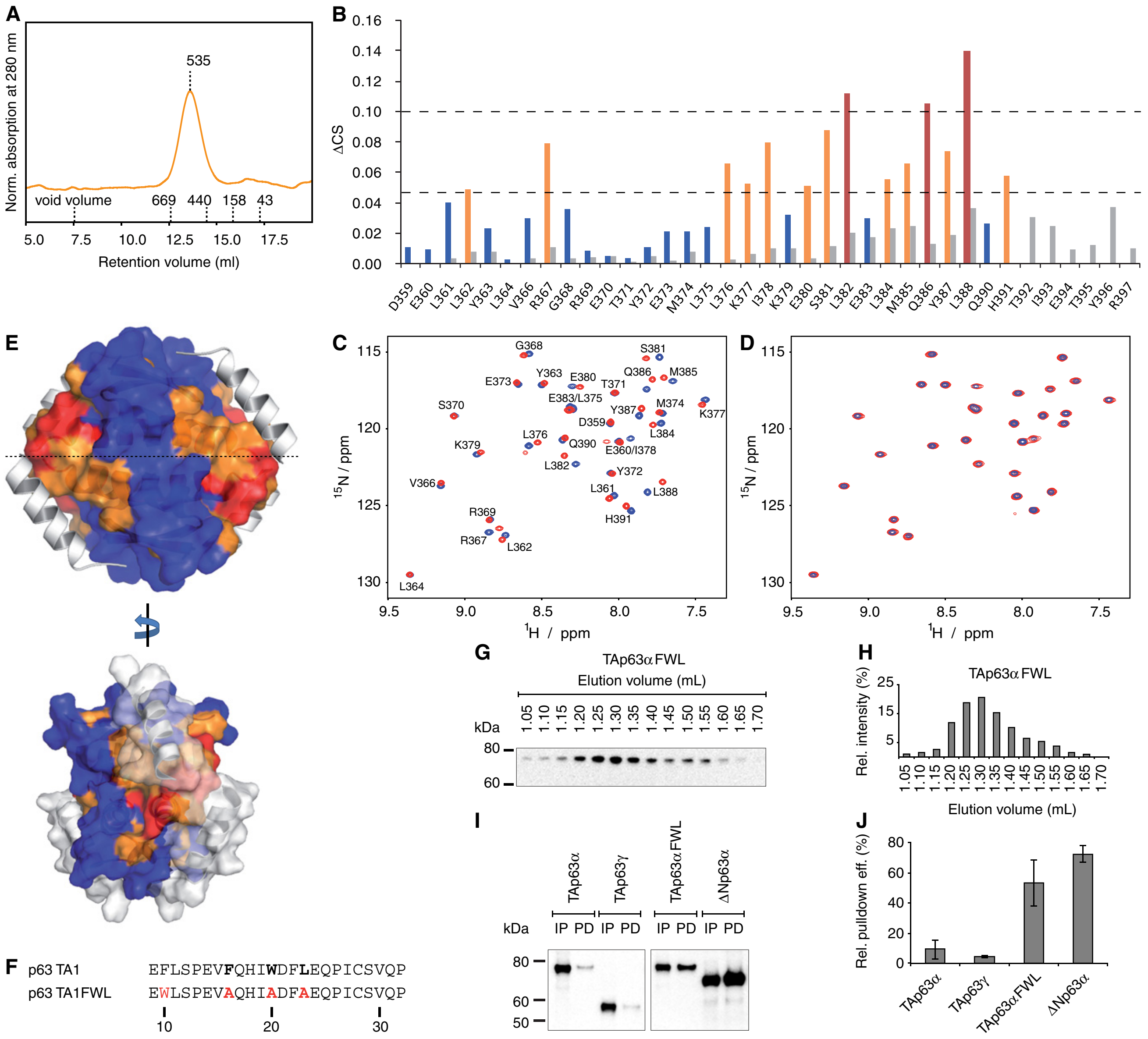


**Figure 6. p63 TA1 Binds to the Oligomerization Domain of p63**

(A) SEC chromatogram of His-MBP-ΔNp63α$^{PPR}$ purified from *E. coli* lacking the last 25 unstructured residues. The apparent molecular weight is indicated. Void volume and elution volumes of globular standard proteins with corresponding molecular weights are shown on the x axis.

(B) Chemical shift perturbations (CSPs) on p63 OD (color coded) and p63 TD (gray) after addition of p63 TA1. OD residues are colored in blue, those with CSP >0.1 ppm are shown in red and those with CSPs >0.05 ppm are shown in orange.

(C and D) [$^{15}$N,$^{1}$H]-TROSY spectra of $^{15}$N-labeled p63 OD in presence (red) and absence (blue) of p63 TA1 (C) and of p63 TA1FWL (D).

(E) Model of p63 TD based on the structure of human p73 TD. Residues are colored according to the code in (B). Helix H2 of the TD (white) lies on top of the TA1-OD interaction interface. H2 is either shown as a ribbon or as a space-filling model.

(F) Sequences of p63 TA1 and p63 TA1FWL with the additional F10W mutation for $UV_{280nm}$-based quantification.

(G and H) Western blot (G) and corresponding bar diagram (H) of SEC elution fractions between 1.05 and 1.70 ml of TAp63αFWL expressed in RRL using an anti-myc antibody.

(I and J) Western blot (I) and corresponding bar diagram (J) of pull-down experiments with TAp63αFWL and ΔNp63α from RRL using immobilized TA domain. Input (IP) and pull-down (PD) for each protein are shown in (I). For comparison, pull-down results for TAp63α and TAp63γ from Figure 5H are shown. Error bars show standard deviation.

contributes to inhibition the most likely mechanism would involve burying these three amino acids to prevent them from interacting with the transcriptional machinery. Mutating these residues to alanine resulted in a complete loss of interaction with the OD, suggesting that they are indeed important for binding, probably by forming one face of a helix (Figures 6D and 6F).

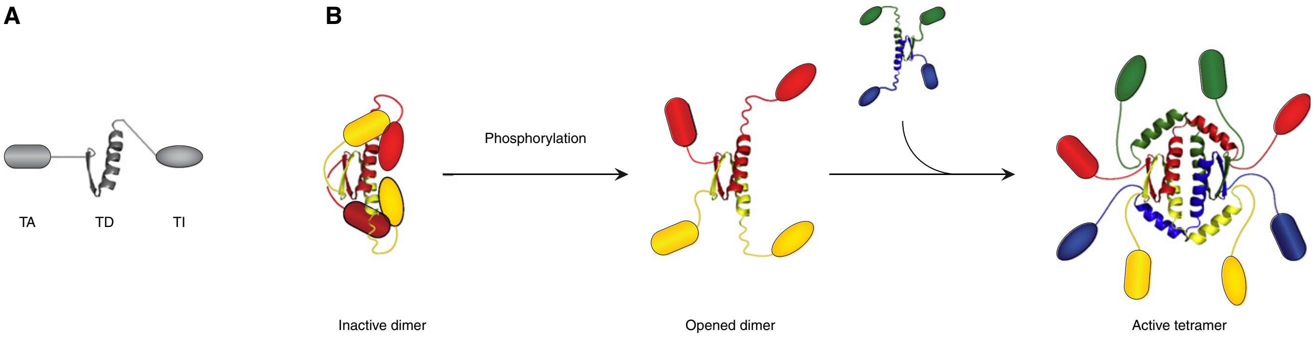


**Figure 7. Model of the Inhibition of TAp63α**

(A) Schematic representation of TAp63α showing the transactivation domain (TA), tetramerization domain (TD) and transactivation inhibitory domain (TI).

(B) Activation of TAp63α requires disruption of the TA-TID-TD interaction network resulting in an open conformation that enables tetramerization. Tetramers are then stabilized by helix H2 in the TD that reaches across the tetramerization interface.

Disrupting the interaction between the TA domain and the OD by mutating these three important residues would expose the binding site for the second helix, potentially leading to the formation of a tetrameric state. Indeed, mutating F16, W20, and L23 in TAp63α to alanine (TAp63αFWL) results in the formation of tetramers (Figures 6G and 6H). Furthermore, pull-down experiments with an external TA domain revealed that the TID is accessible for interaction as expected for an open and tetrameric state (Figures 6I and 6J). These data show that TAp63αFWL behaves similar to ΔNp63α, which lacks the N-terminal part of the TA domain.

## Model of the Structural Regulation of p63's Activity

The data reported here suggest the following model for the regulation of the activity of TAp63α in oocytes. In nonstressed oocytes that are not recruited for ovulation yet, the protein is kept in a dimeric, closed, and inactive conformation (Figure 7). Both the N-terminal transactivation domain and the C-terminal TID are required to form this closed state. The TD plays an essential role as a structural integration domain that interacts with the TA on one side and potentially with the TID on the tetramerization interface. Additional direct contacts between the TA and TI domains have been shown by pull-down experiments (Serber et al., 2002; Straub et al., 2010). The activity of this compact dimeric form is reduced by decreasing its DNA-binding affinity and probably further by burying important amino acids of the TA. Activation requires phosphorylation which triggers a conformational switch that releases the inhibitory interactions, allowing TAp63α to tetramerize and to interact with the transcriptional machinery through its TA. The active tetrameric state is stabilized by the second helix of the TD that reaches across the interface and occupies the binding site of the TA. This model explains how TAp63α can reach high expression levels in oocytes without inducing cell death. Activation of TAp63α, however, is an "irreversible" process that once started leads to the destruction of the oocytes.

## DISCUSSION

The model presented for the regulation of TAp63α's transcriptional activity differs significantly from the model proposed for p53, the most famous member of this protein family. The main regulatory mechanism that determines the activity of p53 seems to be its stability. In nonstressed cells p53 is kept at low concentrations through fast degradation by the E3 ubiquitin ligases Mdm2 and Mdmx (Wade et al., 2010). Oncogenic signals result in a stabilization of p53 leading to an increased cellular concentration. TAp63α on the other hand, is already highly expressed in nonstressed oocytes. DNA damage triggers a conformational change that activates the protein. In contrast to p53, this active form seems to be more susceptible to degradation than the inactive one (Figure 2A). This interpretation is supported by cell culture experiments showing that transcriptionally inactive p63 forms accumulate to much higher concentrations than active ones (Straub et al., 2010). Furthermore, it has been demonstrated that efficient degradation requires an accessible TA domain (Ying et al., 2005). Our NMR data indicate that the three amino acids that are important for binding of p53 to Mdm2 and that are conserved in p63 are most likely not accessible in the inhibited dimeric conformation. While the interaction of p63 with Mdm2 is still discussed controversially, it is obvious that interaction and possible degradation could only occur after activation and the formation of an open state of TAp63α. Regulating the intracellular concentration of TAp63α most likely involves other mechanisms, for example, other E3 ligases. To this end, the Hect-domain E3 ligase ITCH has been shown to ubiquitinate and promote the degradation of p63 (Rossi et al., 2006). Another mechanism that might be specific for the closed dimeric conformation is sumoylation that occurs at the very end of the C terminus of TAp63α where a classical sumoylation site exists. In cell culture experiments mutation of the sumoylation site increased TAp63α's intracellular concentration (Straub et al., 2010). It might therefore be possible that the inhibited dimeric form gets slowly degraded through sumoylation while the active form becomes ubiquitinated by E3 ligases such as ITCH or Mdm2. Degradation of activated TAp63α might occur in oocytes that show a sublethal amount of DNA damage. Measurements of dose-response curves have shown that γ-irradiation with 0.1 Gy results on average in one double-strand break per cell. Most of the oocytes survive this condition with only a small fraction of TAp63α becoming phosphorylated. Irradiation with 0.45 Gy,

however, produces on average ten double-strand breaks per cell and leads to the elimination of virtually all premature oocytes (Suh et al., 2006). Degradation of activated TAp63α might therefore help to establish a certain threshold for the induction of cellular death in oocytes.

A further difference between TAp63α and p53 is that TAp63α is expressed in cells arrested in prophase of meiosis I, therefore presumably inducing only cellular death and not cell cycle arrest (although p63 can in principle induce cell cycle arrest; Guo et al., 2009). From an evolutionary standpoint, quality control of the genetic integrity of oocytes seems to be the original function of the p53 family and cell cycle arrest and tumor suppression evolutionary later developed abilities (Coutandin et al., 2010). This hypothesis is based on the identification of p53-like genes in invertebrate species, for example, *Caenorhabditis elegans* (Pearson and Sanchez Alvarado, 2010). Without renewable tissue and with a relatively short life span, this worm does not require tumor suppression mechanisms. However, its germ cells express a p53-like protein, Cep-1. Both structurally and functionally Cep-1 resembles more closely p63 (Derry et al., 2001; Ou et al., 2007), suggesting that p63 and quality control in germ cells are the ancestral member and function of this protein family.

## EXPERIMENTAL PROCEDURES

### Protein Expression and Purification in *E. coli*

Genes for murine TAp63α, TAp63γ, TAp63αFTL, and TAp63αR (TAp63α carrying the mutation R279H in the DBD; Celli et al., 1999) were cloned into pMAL-c4X vector (New England Biolabs, NEB). All proteins had an additional C-terminal $His_6$-tag. The gene for TAp63α was cloned in the pBH4 vector as well (gift from Wendell Lim laboratory, UCSF) for expression with an N-terminal $His_6$-tag. The gene for murine ΔNp63α lacking the last 25 amino acids ($\Delta Np63\alpha^{PPR}$) was cloned into a modified pMAL vector leading to a fusion protein having an N-terminal $His_6$-tag followed by MBP (His-MBP-$\Delta Np63\alpha^{PPR}$). Proteins were expressed in T7 express competent *E. coli* cells (NEB) and purified using Ni-Sepharose Fast Flow (GE Healthcare) and Amylose resin (NEB) according to standard protocols. Proteins were further purified by size exclusion chromatography (SEC) using a preparative Superose 6 column (GE Healthcare) in 10 mM potassium phosphate buffer (pH 7.6) with 200 mM NaCl. All following experiments were performed in this storage buffer if not denoted differently.

### Protein Expression in Rabbit Reticulocyte Lysate (RRL)

N-terminally myc-tagged murine TAp63α, TAp63αFTL, as well as TAp63α and TAp63αFTL lacking helix H2 in the TD (TAp63αΔHelix, TAp63αFTLΔHelix), TAp63α triple mutant F16A, W20A, L23A (TAp63αFWL), TAp63α double mutants L384A/M385A (TAp63αLM) and M374Q/I378R (TAp63αMI), and TAp63α mutant L384A (TAp63αL), TAp63γ, and ΔNp63α were expressed from pcDNA3.1 vector in RRL as described (Straub et al., 2010). Proteins were used for SEC analysis with a Superose 6 PC 3.2/30 column (GE Healthcare).

### Size Exclusion Chromatography

SEC of recombinant proteins expressed in *E. coli* was performed at 16°C using a Superose 6 10/300 GL column (GE Healthcare), calibrated using Blue Dextran 2000, Thyroglobulin (669 kDa), Ferritin (440 kDa), Aldolase (158 kDa), and Ovalbumin (43 kDa) (GE Healthcare) (Figures 1A and 5A; Figure S2A). All other SEC experiments were performed at 4°C using a Superose 6 PC 3.2/30 column (GE Healthcare) (flow rate 0.05 ml/min; fraction size 50 µl). Relevant SEC fractions were analyzed by western blotting.

### Cysteine Accessibility Assay

The assay followed a protocol described previously (Lambert et al., 2009), with modifications. MBP-TAp63α, MBP-TAp63αFTL, BSA and storage buffer were incubated with 2.4 µM Maleimide-$PEG_2$-Biotin (Pierce) for 1 hr at RT. The reaction was stopped by adding 44 µM cysteine followed by 1 hr incubation. Samples were immobilized for 1 hr on a 96 well Nickel coated plate (Pierce). Wells were then washed three times with PBS, blocked for 1 hr with 5% skim milk in PBS, probed with either Avidin-HRP conjugate (Pierce) or HPR conjugated anti-MBP antibody (NEB) for 50 min, washed seven times with PBS and processed as described. Intensities were averaged over three wells. The ratio of Avidin-HRP conjugate and HRP conjugated anti-MBP antibody signal intensities corresponds to the cysteine accessibility. The ratio of MBP-TAp63α was set to 100%. Each experiment was performed three times and the results were averaged.

### Multiangle Light Scattering

SEC-MALS experiments were performed at room temperature using a Superdex 200 5/150 GL column (GE Healthcare) at a flow rate of 0.3 ml/min. Elution of 80 µl of purified proteins of ~1 mg/ml concentration was detected using an Optilab rEX Refractive Index Detector and a Dawn Heleos II at a Laser wavelength of 658 nm (Wyatt Technology) to determine the weight average molar mass $\overline{M}_W$ of peak locations. Data were processed using ASTRA software package 5.3.4.11 (Wyatt Technology).

### Mice and Irradiation

Animal care and handling were performed according to the guidelines set by the World Health Organization (Geneva, Switzerland). Five-day-old (P5) female CD-1 mice were purchased from Charles River Laboratories. Animals were divided into two groups, NIRR (nonirradiated) and IRR (irradiated). IRR mice were exposed to 0.52 Gy of whole-body γ-irradiation on a rotating turntable in a $^{137}$Cs irradiator, at a dose rate of 2.387 Gy/min. Ovaries of both groups were harvested after 6 hr.

### Analysis of Murine Ovary Extracts

Sixteen ovaries of NIRR or IRR mice were lysed by mechanical force in 50 mM sodium phosphate, pH = 7.2, 150 mM NaCl, 0.1% Triton X-100, EDTA free protease inhibitor cocktail (Roche) and phosphatase inhibitor cocktail (Roche) in a total volume of 70 µl. After centrifugation at 20,000 × g for 15 min at 4°C the supernatant was injected in a Superose 6 PC 3.2/30 column (GE Healthcare) equilibrated with 50 mM sodium phosphate, 100 mM NaCl, EDTA free protease inhibitor cocktail and phosphatase inhibitor cocktail at 4°C and eluted as described above. Collected fractions were separated using 10% Bis-Tris NuPAGE gels (Invitrogen) in MOPS buffer at 4°C and subsequently transferred on a Hybond-P membrane (GE Healthcare) using a XCell II blot module (Invitrogen). Blots were then blocked with 5% skim milk in TBS buffer containing 0.1% Tween-20 and probed over night at 4°C with 4A4 (Suh et al., 2006) antibody. Detection was performed using goat anti-mouse IgG peroxide conjugate (Sigma Aldrich). Blots were quantified using Biometra BioDocAnalyze 2.0 software.

### Fluorescence Anisotropy

FA experiments were performed at room temperature using a Jasco spectrofluorometer FP-6500 (Jasco Labortechnik). MBP-TAp63α, MBP-TAp63αFTL, and MBP-TAp63γ purified from *E. coli* were added to 15 nM of 5′-fluorescein-tagged dsDNA with the sequence of the p21 promoter response element (5′-GGCAGGAACATGTCCCAACATGTTGAGCCG-3′) in final monomeric concentrations between 0.1 and 15 µM in a total volume of 500 µl. Protein and DNA were incubated for 30 min at room temperature before being measured with excitation at 488 nm and emission at 516 nm. Each experimental series was repeated three times (MBP-TAp63α and MBP-TAp63αFTL) or twice (MBP-TAp63γ) and averaged. Data were analyzed using the software Origin (OriginLab Corporation). Dissociation constants $K_D$ were calculated by fitting the data to the equation shown below resembling a two-binding-site model:

$$Y = \frac{A^{C1}K_2[P] + A^{C2}[P]^2 + K_1K_2A^D}{K_1K_2 + K_2[P] + [P]^2},$$

with Y being the measured FA, $A^{C1}$, $A^{C2}$, and $A^D$ the FA values of a complex with one p63 oligomer bound to DNA, of a complex with two p63 oligomers

bound to DNA and of the free DNA, respectively, [P] the monomeric concentration of the protein and $K_1$ and $K_2$ the two binding constants. For MBP-TAp63αFTL and MBP-TAp63γ the $K_D$ values for the second binding site were 47.1 ± 115.7 μM and 20.8 ± 18.0 μM, respectively, suggesting that they represent unspecific binding. For MBP-TAp63α a negative $K_D$ value of −0.021 ± 0.02 μM was obtained which we cannot interpret at the moment. Control experiments were performed with MBP-TAp63α, MBP-TAp63αFTL, MBP-TAp63γ, and MBP-TAp63αR as described. 5′-Fluorescein-tagged dsDNA (300 nM) with either a p63 binding sequence designed on the basis of a SELEX (Ortt and Sinha, 2006) screening (5′-CCTATTCTAGACATGTGAG GACATGTCGATACTTATTCC-3′) or a random sequence (5′-CGAGTTGTAA GTCGAATTGATACCATAATGCACTACACG-3′) was used.

### λ-Phosphatase Treatment

Thirty ovaries of IRR mice were lysed in 50 mM sodium phosphate (pH 7.2) 150 mM NaCl, EDTA free protease inhibitor cocktail (Roche), and 1.25 mM $MnCl_2$ in a final volume of 120 μl as described above. To one half of the lysate 15 μl of λ-Protein Phosphatase (NEB), to the other 15 μl of λ-Protein Phosphatase storage buffer were added. Both samples were incubated at 30°C for 45 min. Samples were centrifuged and analyzed by SEC and western blotting as described above.

### Cell Culture Experiments

Transactivation assays of p63 isoforms and mutants as well as corresponding western blot analyses were performed as described previously (Straub et al., 2010). Each measurement was carried out in triplicates and averaged.

### Pull-Down Experiments

Pull-down experiments with p63 isoforms and mutants expressed in RRL and immobilized GST-TID (aa 569–616) or GST-TA (aa 1–136) as well as corresponding western blot analyses were performed as described previously (Straub et al., 2010). Each experiment was performed three times and the results were averaged.

### Western Blotting

Western blot analysis was performed as described previously (Straub et al., 2010).

### NMR Titrations

Human p63 OD (358-391) and human p63 TD (358-416) was cloned and expressed as described (Coutandin et al., 2009). Peptides (p63 TA1 [9-32] and p63 TA1FWL with the triple mutation F16A, W20A, L23A and mutation F10W) were ordered from Genscript (Piscataway, NJ, USA). NMR titration experiments (up to 15-fold excess of peptide) of $^{15}$N-labeled protein samples (100 μM) in 25 mM HEPES, 50 mM Arg, 50 mM Glu (pH 7.5) were performed at 303 K on Bruker Avance spectrometers operating at proton frequencies of 900 and 800 MHz. Spectra were analyzed with UCSF SPARKY 3.114. Structural Models of human p63 OD based on the structure of human p73 TD (PDB ID 2KBY) are illustrated using Pymol 1.0.

### Glutaraldehyde Crosslinks

MBP-TAp63α, MBP-TAp63αFTL, and MBP-TAp63γ purified from *E. coli* at concentrations of 0.5, 2, and 10 μM and TAp63α at concentrations of 0.5, 2, and 4 μM were crosslinked with 0.125% (w/v) glutaraldehyde (Merck). Samples were incubated at 16°C for 3, 20, and 68 min and immediately analyzed by SDS-PAGE using 4%–12% Bis-Tris NuPAGE gels (Invitrogen) followed by Coomassie staining.

### LILBID Mass Spectrometry

Mass spectra were recorded using the previously described LILBID-MS method (Hoffmann et al., 2009; Morgner et al., 2007). MBP-TAp63α expressed in *E. coli* was dialyzed over night against 10 mM potassium phosphate (pH 7.6) without NaCl.

### Analytical Ultracentrifugation

Sedimentation of samples at monomer concentrations of 1–8 μM at 4°C in 20 mM potassium phosphate pH 7.5, 200 mM NaCl, 2 mM dithiothreitol was monitored using absorbance (at 280 nm) and interference optics in a Beckman XL-I Analytical Ultracentrifuge equipped with a Ti-50 rotor. Sedimentation velocity experiments were performed at a rotor speed of 45,000 rpm, while equilibrium experiments were carried out employing rotor speeds of 7000 and 9000 rpm once equilibrium conditions had been established. Analysis of velocity data was performed using SEDFIT (Lebowitz et al., 2002), while HETEROANALYSIS (Cole, 2004) and ULTRASPIN (www.ultraspin.mrc-cpe.cam.ac.uk) were employed for global fitting of equilibrium scans (per protein: three concentrations and two speeds) to simple, nonassociation models in order to determine the average weight of the sedimenting species.

### In Vitro Phosphorylation

Radiolabeling of MBP-TAp63α was performed as described previously (Schleiff et al., 2003). The phosphorylation profile was visualized by Coomassie staining and autoradiography.

## SUPPLEMENTAL INFORMATION

Supplemental Information includes five figures and one table and can be found with this article online at doi:10.1016/j.cell.2011.01.013.

## ACKNOWLEDGMENTS

The research was funded by the DFG (DO 545/2-1), EU-Grant EPISTEM (LSHB-CT-019067), the Centre for Biomolecular Magnetic Resonance (BMRZ), and the Cluster of Excellence Frankfurt (Macromolecular Complexes). F.M. acknowledges support by ANR and ERC. D.C. was supported by a Boehringer Ingelheim Fonds PhD Fellowship. The Structural Genomics Consortium is a registered charity (number 1097737) that receives funds from the Canadian Institutes for Health Research, the Canadian Foundation for Innovation, Genome Canada through the Ontario Genomics Institute, GlaxoSmithKline, Karolinska Institutet, the Knut and Alice Wallenberg Foundation, the Ontario Innovation Trust, the Ontario Ministry for Research and Innovation, Merck & Co., Inc., the Novartis Research Foundation, the Swedish Agency for Innovation Systems, the Swedish Foundation for Strategic Research, and the Wellcome Trust. J. Hannewald is an employee of Merck KGaA, Darmstadt.

Received: June 18, 2010
Revised: November 5, 2010
Accepted: December 17, 2010
Published: February 17, 2011

## REFERENCES

Brandt, T., Petrovich, M., Joerger, A.C., and Veprintsev, D.B. (2009). Conservation of DNA-binding specificity and oligomerisation properties within the p53 family. BMC Genomics *10*, 628.

Brooks, C.L., and Gu, W. (2006). p53 ubiquitination: Mdm2 and beyond. Mol. Cell *21*, 307–315.

Burge, S., Teufel, D.P., Townsley, F.M., Freund, S.M., Bycroft, M., and Fersht, A.R. (2009). Molecular basis of the interactions between the p73 N terminus and p300: effects on transactivation and modulation by phosphorylation. Proc. Natl. Acad. Sci. USA *106*, 3142–3147.

Celli, J., Duijf, P., Hamel, B.C., Bamshad, M., Kramer, B., Smits, A.P., Newbury-Ecob, R., Hennekam, R.C., Van Buggenhout, G., van Haeringen, A., et al. (1999). Heterozygous germline mutations in the p53 homolog p63 are the cause of EEC syndrome. Cell *99*, 143–153.

Chan, W.M., Siu, W.Y., Lau, A., and Poon, R.Y. (2004). How many mutant p53 molecules are needed to inactivate a tetramer? Mol. Cell. Biol. *24*, 3536–3551.

Chene, P. (2001). The role of tetramerization in p53 function. Oncogene *20*, 2611–2617.

Cole, J.L. (2004). Analysis of heterogeneous interactions. Methods Enzymol. *384*, 212–232.

Coutandin, D., Löhr, F., Niesen, F., Ikeya, T., Weber, T., Schäfer, B., Bullock, A., Yang, A., Güntert, P., Knapp, S., et al. (2009). Conformational stability

and activity of p73 require a second helix in the tetramerization domain. Cell Death Differ. *16*, 1582–1589.

Coutandin, D., Der Ou, H., Lohr, F., and Dotsch, V. (2010). Tracing the protectors path from the germ line to the genome. Proc. Natl. Acad. Sci. USA *107*, 15318–15325.

Davison, T.S., Nie, X., Ma, W., Lin, Y., Kay, C., Benchimol, S., and Arrowsmith, C.H. (2001). Structure and functionality of a designed p53 dimer. J. Mol. Biol. *307*, 605–617.

De Laurenzi, V., Catani, M., Terrinoni, A., Corazzari, M., Melino, G., Costanzo, A., Levrero, M., and Knight, R.A. (1999). Additional complexity in p73: induction by mitogens in lymphoid cells and identification of two new splicing variants epsilon and zeta. Cell Death Differ. *6*, 389–390.

Derry, W.B., Putzke, A.P., and Rothman, J.H. (2001). Caenorhabditis elegans p53: role in apoptosis, meiosis, and stress resistance. Science *294*, 591–595.

Guo, X., Keyes, W.M., Papazoglu, C., Zuber, J., Li, W., Lowe, S.W., Vogel, H., and Mills, A.A. (2009). TAp63 induces senescence and suppresses tumorigenesis in vivo. Nat. Cell Biol. *11*, 1451–1457.

Hoffmann, J., Schmidt, T.L., Heckel, A., and Brutschy, B. (2009). Probing the limits of liquid droplet laser desorption mass spectrometry in the analysis of oligonucleotides and nucleic acids. Rapid Commun. Mass Spectrom. *23*, 2176–2180.

Horikoshi, N., Usheva, A., Chen, J., Levine, A.J., Weinmann, R., and Shenk, T. (1995). Two domains of p53 interact with the TATA-binding protein, and the adenovirus 13S E1A protein disrupts the association, relieving p53-mediated transcriptional repression. Mol. Cell. Biol. *15*, 227–234.

Jeffrey, P.D., Gorina, S., and Pavletich, N.P. (1995). Crystal structure of the tetramerization domain of the p53 tumor suppressor at 1.7 angstroms. Science *267*, 1498–1502.

Joerger, A.C., Rajagopalan, S., Natan, E., Veprintsev, D.B., Robinson, C.V., and Fersht, A.R. (2009). Structural evolution of p53, p63, and p73: implication for heterotetramer formation. Proc. Natl. Acad. Sci. USA *106*, 17705–17710.

Kaghad, M., Bonnet, H., Yang, A., Creancier, L., Biscan, J.C., Valent, A., Minty, A., Chalon, P., Lelias, J.M., Dumont, X., et al. (1997). Monoallelically expressed gene related to p53 at 1p36, a region frequently deleted in neuroblastoma and other human cancers. Cell *90*, 809–819.

Kussie, P.H., Gorina, S., Marechal, V., Elenbaas, B., Moreau, J., Levine, A.J., and Pavletich, N.P. (1996). Structure of the MDM2 oncoprotein bound to the p53 tumor suppressor transactivation domain. Science *274*, 948–953.

Lambert, J.M., Gorzov, P., Veprintsev, D.B., Soderqvist, M., Segerback, D., Bergman, J., Fersht, A.R., Hainaut, P., Wiman, K.G., and Bykov, V.J. (2009). PRIMA-1 reactivates mutant p53 by covalent binding to the core domain. Cancer Cell *15*, 376–388.

Lebowitz, J., Lewis, M.S., and Schuck, P. (2002). Modern analytical ultracentrifugation in protein science: a tutorial review. Protein Sci. *11*, 2067–2079.

Lee, W., Harvey, T.S., Yin, Y., Yau, P., Litchfield, D., and Arrowsmith, C.H. (1994). Solution structure of the tetrameric minimum transforming domain of p53. Nat. Struct. Biol. *1*, 877–890.

Lu, H., and Levine, A.J. (1995). Human TAFII31 protein is a transcriptional coactivator of the p53 protein. Proc. Natl. Acad. Sci. USA *92*, 5154–5158.

McGrath, J.A., Duijf, P.H., Doetsch, V., Irvine, A.D., de Waal, R., Vanmolkot, K.R., Wessagowit, V., Kelly, A., Atherton, D.J., Griffiths, W.A., et al. (2001). Hay-Wells syndrome is caused by heterozygous missense mutations in the SAM domain of p63. Hum. Mol. Genet. *10*, 221–229.

Mills, A.A., Zheng, B., Wang, X.J., Vogel, H., Roop, D.R., and Bradley, A. (1999). p63 is a p53 homologue required for limb and epidermal morphogenesis. Nature *398*, 708–713.

Morgner, N., Kleinschroth, T., Barth, H.D., Ludwig, B., and Brutschy, B. (2007). A novel approach to analyze membrane proteins by laser mass spectrometry: from protein subunits to the integral complex. J. Am. Soc. Mass Spectrom. *18*, 1429–1438.

Ortt, K., and Sinha, S. (2006). Derivation of the consensus DNA-binding sequence for p63 reveals unique requirements that are distinct from p53. FEBS Lett. *580*, 4544–4550.

Ou, H.D., Löhr, F., Vogel, V., Mäntele, W., and Dötsch, V. (2007). Structural evolution of C-terminal domains in the p53 family. EMBO J. *26*, 3463–3473.

Pearson, B.J., and Sanchez Alvarado, A. (2010). A planarian p53 homolog regulates proliferation and self-renewal in adult stem cell lineages. Development *137*, 213–221.

Rossi, M., Aqeilan, R.I., Neale, M., Candi, E., Salomoni, P., Knight, R.A., Croce, C.M., and Melino, G. (2006). The E3 ubiquitin ligase Itch controls the protein stability of p63. Proc. Natl. Acad. Sci. USA *103*, 12753–12758.

Schleiff, E., Jelic, M., and Soll, J. (2003). A GTP-driven motor moves proteins across the outer envelope of chloroplasts. Proc. Natl. Acad. Sci. USA *100*, 4604–4609.

Schmale, H., and Bamberger, C. (1997). A novel protein with strong homology to the tumor suppressor p53. Oncogene *15*, 1363–1367.

Senoo, M., Pinto, F., Crum, C.P., and McKeon, F. (2007). p63 Is essential for the proliferative potential of stem cells in stratified epithelia. Cell *129*, 523–536.

Senoo, M., Seki, N., Ohira, M., Sugano, S., Watanabe, M., Inuzuka, S., Okamoto, T., Tachibana, M., Tanaka, T., Shinkai, Y., et al. (1998). A second p53-related protein, p73L, with high homology to p73. Biochem. Biophys. Res. Commun. *248*, 603–607.

Serber, Z., Lai, H.C., Yang, A., Ou, H.D., Sigal, M.S., Kelly, A.E., Darimont, B.D., Duijf, P.H., Van Bokhoven, H., McKeon, F., et al. (2002). A C-terminal inhibitory domain controls the activity of p63 by an intramolecular mechanism. Mol. Cell. Biol. *22*, 8601–8611.

Straub, W.E., Weber, T.A., Schäfer, B., Candi, E., Durst, F., Ou, H.D., Rajalingam, K., Melino, G., and Dötsch, V. (2010). The C-terminus of p63 contains multiple regulatory elements with different functions. Cell Death Dis. *1*, e5. 10.1038/cddis.2009.1.

Suh, E.K., Yang, A., Kettenbach, A., Bamberger, C., Michaelis, A.H., Zhu, Z., Elvin, J.A., Bronson, R.T., Crum, C.P., and McKeon, F. (2006). p63 protects the female germ line during meiotic arrest. Nature *444*, 624–628.

Thut, C.J., Chen, J.L., Klemm, R., and Tjian, R. (1995). p53 transcriptional activation mediated by coactivators TAFII40 and TAFII60. Science *267*, 100–104.

Trink, B., Okami, K., Wu, L., Sriuranpong, V., Jen, J., and Sidransky, D. (1998). A new human p53 homologue. Nat. Med. *4*, 747–748.

Wade, M., Wang, Y.V., and Wahl, G.M. (2010). The p53 orchestra: Mdm2 and Mdmx set the tone. Trends Cell Biol. *20*, 299–309.

Yang, A., Kaghad, M., Wang, Y., Gillett, E., Fleming, M.D., Dotsch, V., Andrews, N.C., Caput, D., and McKeon, F. (1998). p63, a p53 homolog at 3q27-29, encodes multiple products with transactivating, death-inducing, and dominant-negative activities. Mol. Cell *2*, 305–316.

Yang, A., Schweitzer, R., Sun, D., Kaghad, M., Walker, N., Bronson, R.T., Tabin, C., Sharpe, A., Caput, D., Crum, C., et al. (1999). p63 is essential for regenerative proliferation in limb, craniofacial and epithelial development. Nature *398*, 714–718.

Yang, A., Walker, N., Bronson, R., Kaghad, M., Oosterwegel, M., Bonnin, J., Vagner, C., Bonnet, H., Dikkes, P., Sharpe, A., et al. (2000). p73-deficient mice have neurological, pheromonal and inflammatory defects but lack spontaneous tumours. Nature *404*, 99–103.

Ying, H., Chang, D.L., Zheng, H., McKeon, F., and Xiao, Z.X. (2005). DNA-binding and transactivation activities are essential for TAp63 protein degradation. Mol. Cell. Biol. *25*, 6154–6164.

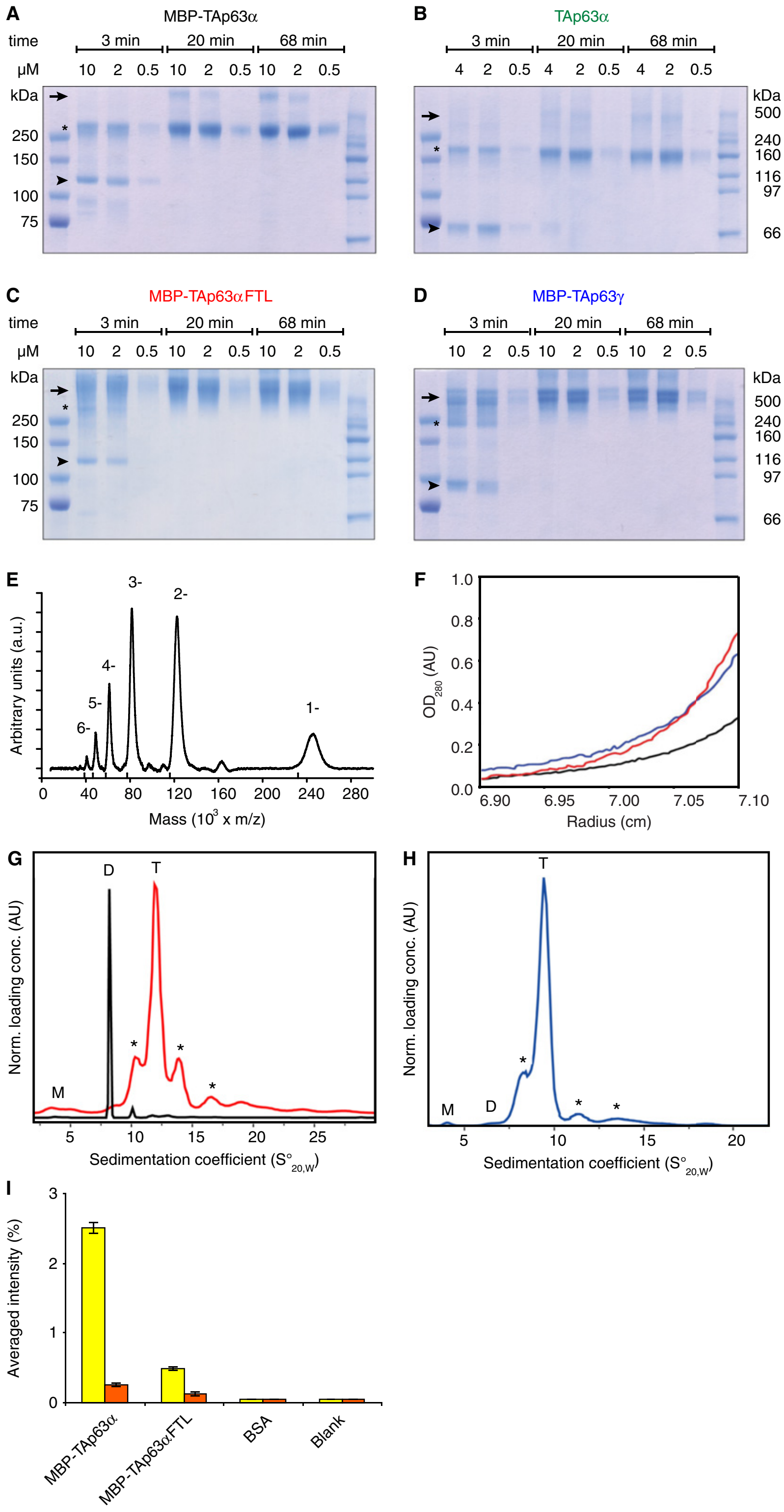


**Figure S1. TAp63α Forms Inactive, Closed Dimers, whereas Active Forms Are Tetrameric and Open, Related to Figure 1**
(A–D) Coomassie stained SDS-PAGE analyses of MBP-TAp63α (A), TAp63α (B), MBP-TAp63αFTL (C) and MBP-TAp63γ (D) purified from *E. coli* cross linked with glutaraldehyde. Different concentrations of protein and different reaction times were used as indicated. Protein bands corresponding to monomeric, dimeric and higher oligomeric proteins are labeled by arrowheads, asterisks and arrows, respectively.

(E) LILBID anion spectrum of MBP-TAp63α. The spectrum was recorded using a protein concentration of ~10 μM and averaged over 200 droplets. The ruler indicates the charge distribution of a 232 kDa molecule. Laser energy was adjusted to 8mJ/pulse. Masses correspond to a dimeric protein with charges between 1 and 6. A small peak for the triple charged tetramer is seen at 155 × $10^3$ m/z (beginning of the peak).

(F) AUC sedimentation equilibrium absorbance scans (at 280 nm) of MBP-TAp63α (black), MBP-TAp63α FTL (red) and MBP-TAp63γ (blue). A protein concentration of 4 μM and a speed of 7000 rpm was used.

(G and H) Species distributions of MBP-TAp63α (black) and MBP-TAp63αFTL (red), as well as of MBP-TAp63γ (blue) (H) according to their sedimentation coefficients, determined by AUC sedimentation velocity at 45,000 rpm. A protein concentration of 4 μM was used. The positions of monomers (M), dimers (D) and tetramers (T) are denoted in the graph. Additional peaks visible for MBP-TAp63αFTL and MBP-TAp63γ, as denoted by asterisks, indicate low concentration of additional species (trimer, pentamer, etc.). Comparison of the apparent sedimentation coefficient and frictional ratio of tetrameric MBP-TAp63αFTL and MBP-TAp63γ with the wild-type dimer supports an open conformation of the tetrameric form.

(I) Bar diagram showing absolute signal intensities of the detection of MBP (yellow) and maleimide-biotin conjugate covalently linked to accessible cysteines (orange) of MBP-TAp63α and MBP-TAp63αFTL purified from *E. coli*, BSA and as negative control buffer, averaged over three experiments. Error bars show standard deviation.

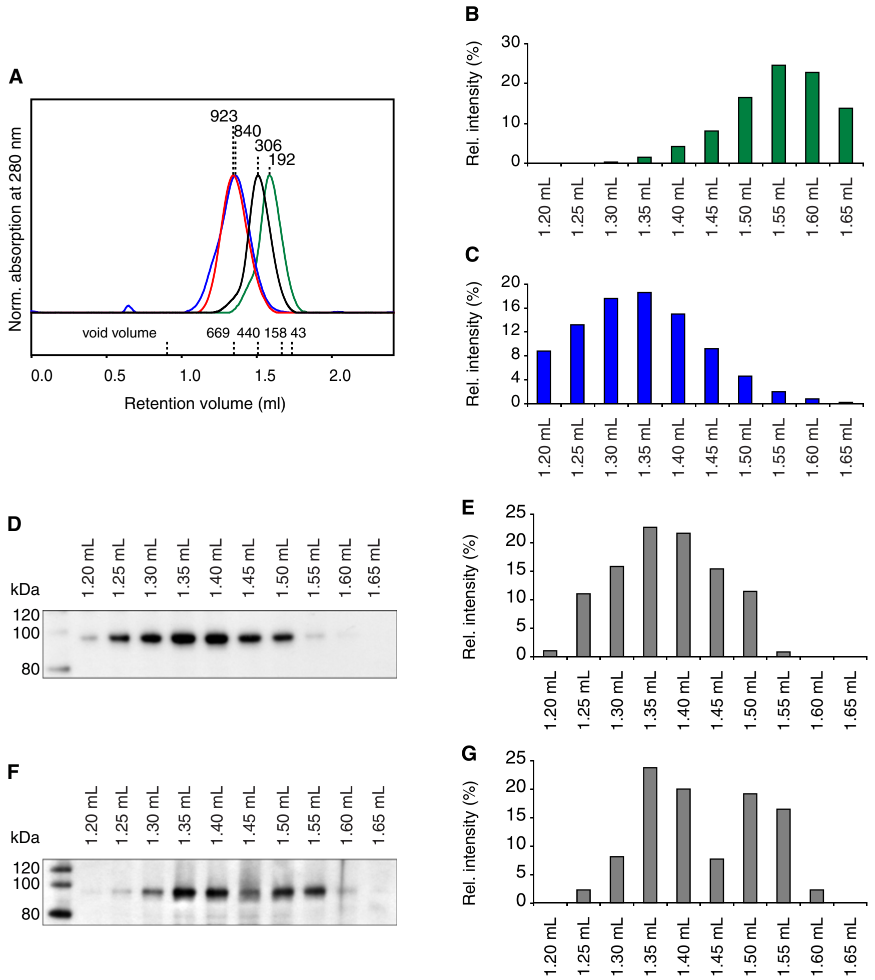

**Figure S2. Dilution of MBP-TAp63γ Leads to a Shift of the Tetramerization Equilibrium toward Dimers, Related to Figure 2**

(A) SEC chromatogram of MBP-TAp63α (black), MBP-TAp63αFTL (red), MBP-TAp63γ (blue) and TAp63α (green) purified from *E. coli* indicating the amount of overlap of the tetramer and dimer fractions on a Superose 6 PC 3.2/30 column. Apparent molecular weights are indicated. Void volume and elution volumes of globular standard proteins with corresponding molecular weights are shown on the *x* axis. The chromatogram reveals that p63 forms show the same relative elution profiles on a Superose 6 PC 3.2/30 column as on a Superose 6 10/300 GL column (results shown in Figure 5B). Deviations in the predicted masses of the peaks between both columns are due to the lower resolution of the Superose 6 PC 3.2/30 column.

(B and C) Bar diagrams showing the intensities of TAp63α (B) and MBP-TAp63γ (C) relative to the respective total intensity for the SEC chromatogram shown in (A). Each value represents a 50 μl fraction between 1.2 and 1.65 ml elution volume. The diagram in (B) displays a maximum at ~1.55 ml (dimer fraction) and the diagram in (C) at ~1.35 ml (tetramer fraction) elution volume. The concentration of both proteins was approximately 5 μM.

(D) Western blot of 50 μl SEC elution fractions between 1.2 and 1.65 ml of 100 nM MBP-TAp63γ purified from *E. coli*.

(E) Bar diagrams showing the MBP-TAp63γ signal intensities relative to the sum of all intensities of the Western blot in (D).

(F and G) Western blot of 50 μl SEC elution fractions between 1.2 and 1.65 ml of 30 nM MBP-TAp63γ (F) and corresponding bar diagram (G).

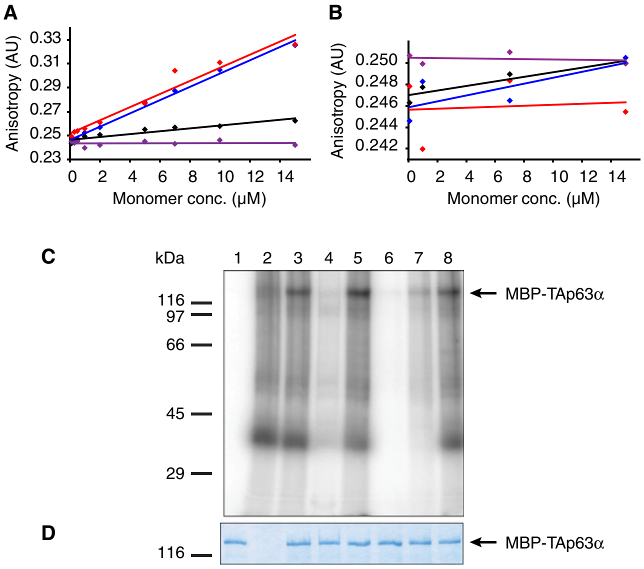


| | 1 | 2 | 3 | 4 | 5 | 6 | 7 | 8 |
|---|---|---|---|---|---|---|---|---|
| 10 μCi [γ-$^{32}$P-] ATP | + | + | + | + | + | + | + | + |
| MBP-TAp63α | + | - | + | + | + | + | + | + |
| IRR ovary lysate | - | + | + | + | + | + | + | + |
| 5 U λ-PP | - | - | - | + | - | - | - | - |
| Phosphatase inhibitors | - | - | - | - | + | - | - | - |
| 100 μM ATP | - | - | - | - | - | + | - | - |
| 100 μM GTP | - | - | - | - | - | - | + | - |
| 100 μM ADP | - | - | - | - | - | - | - | + |

**Figure S3. DNA Binding of TAp63 Is Specific, whereas λ-Phosphatase Treatment Leads to Complete Dephosphorylation, Related to Figure 3**

(A and B) Binding of MBP-TAp63α (black dots), MBP-TAp63αFTL (red dots), MBP-TAp63γ (blue dots) and MBP-TAp63αR (purple dots) purified from *E. coli* to 300 nM of SELEX DNA (see Methods section) (A) or to a random sequence (see Methods section) (B) using fluorescence anisotropy. In (A) all TAp63 forms except MBP-TAp63αR bind to the SELEX DNA, with MBP-TAp63αFTL and MBP-TAp63γ showing higher affinity than MBP-TAp63α. In (B) none of the TAp63 forms binds to the random sequence.

(C) In vitro phosphorylation assay in which MBP-TAp63α purified from *E. coli* (lanes 1; 3-8) was incubated in the absence (lane 1) or presence (lanes 2-8) of lysed IRR ovaries (lane 2) for 30 min at RT with 10 μCi [γ-$^{32}$P]-ATP. The reaction was challenged by 5 U λ-phosphatase (λ-PP) (lane 4), phosphatase inhibitors (Lane 5), 100 μM ATP (lane 6), 100 μM GTP (lane 7) or 100 μM ADP (lane 8). Reactions were stopped by SDS sample buffer and the samples were analyzed on 10% SDS-PAGE. The phosphorylation profile was visualized by autoradiography.

(D) Coomassie staining of the gel corresponding to (C) showing MBP-TAp63α as loading control.

Cell

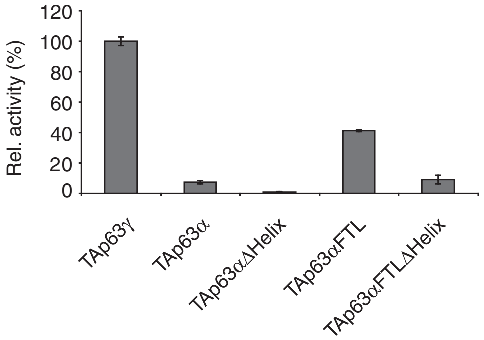


**Figure S4. The Second Helix H2 in the TD Is Essential for the Formation of Stable Tetramers, Related to Figure 4**

Transcriptional activities on the p21 promoter in SAOS2 cells of TAp63γ, TAp63α, TAp63α without Helix H2 (TAp63αΔHelix), TAp63αFTL and TAp63αFTL without Helix H2 (TAp63αFTLΔHelix) with the activity of TAp63γ set to 100%. Experiments were performed in triplicate. Error bars show standard deviation.

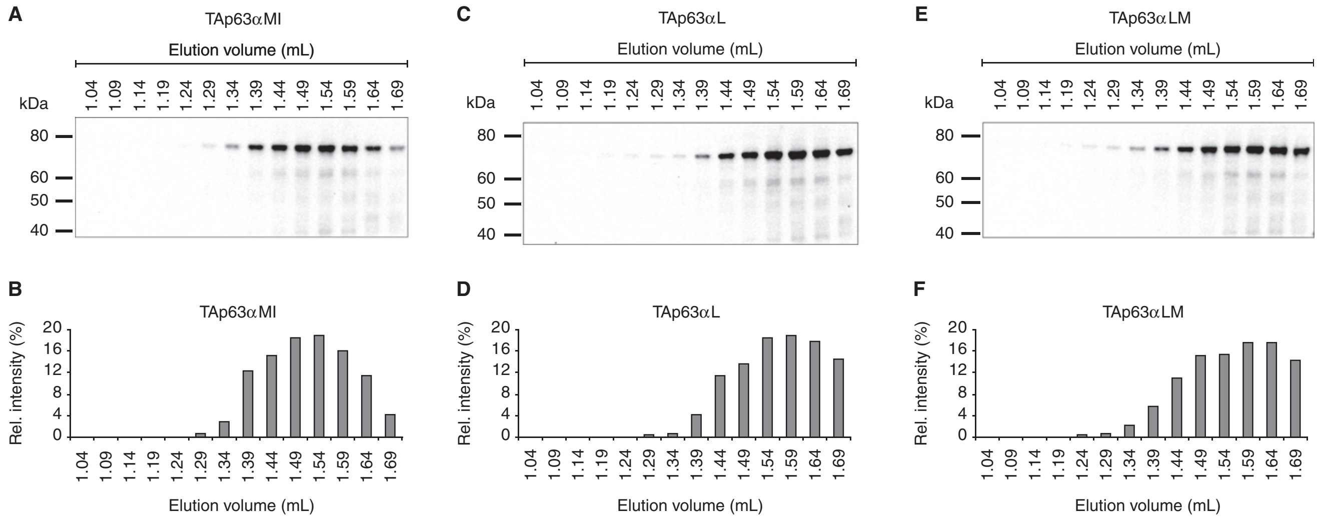


**Figure S5. SEC Analysis of Mutations in the Tetramerization Interface of the TD of TAp63α, Related to Figure 5**

(A and B) Western blot analysis of SEC elution fractions from 1.04 to 1.69 ml of TAp63αMI expressed in rabbit reticulocyte lysate using an anti-myc antibody (A) and bar diagram of the Western blot intensities (B).

(C–F) Corresponding data and analysis of TAp63αL and TAp63αLM are shown in (C and D) and (E and F), respectively. The introduction of the M374Q/I378R dimer mutation in the center of the tetramerization interface of TAp63αMI leads to the destabilization of the inhibitory TA-TID interaction. This results in a dimeric protein with a more opened conformation in which both the TA domain and the TID are at least partially accessible for interactions with the corresponding external interaction partner in pull-down assays. Mutations introduced at the edges of the tetramerization interface (TAp63αLM and TAp63αL) do not affect the interaction between TID and TA domain suggesting the involvement of specific areas of the TD in the inhibitory mechanism mediated by the TID.
